# Use of Attitude and Heading Reference System (AHRS) to Analyze the Impact of Safety Nets on the Accelerations Occurring in the Human Body During a Collision

**DOI:** 10.3390/s24237431

**Published:** 2024-11-21

**Authors:** Mariusz Gołkowski, Jerzy Kwaśniewski, Maciej Roskosz, Paweł Mazurek, Szymon Molski, Józef Grzybowski

**Affiliations:** 1 CBR Rock Master, 30-079 Krakow, Poland; mariusz.golkowski@rockmaster.eu; 2 Department of Machinery Engineering and Transport, Faculty of Mechanical Engineering and Robotics, AGH University of Krakow, 30-059 Krakow, Poland; kwasniew@agh.edu.pl (J.K.); mroskosz@agh.edu.pl (M.R.); molski@agh.edu.pl (S.M.); 3 Department of Avionics and Control, Rzeszów University of Technology, Al, Powstańców Warszawy 8, 35–959 Rzeszów, Poland; aviog@prz.edu.pl

**Keywords:** safety, safety nets, attitude and heading reference system (AHRS), microelectromechanical systems (MEMS), acceleration measurement

## Abstract

The article presents accelerations occurring in the human body when falling onto a safety net. An attitude and heading reference system (AHRS) consists of sensors on three axes that provide attitude information for objects, including pitch, roll, and yaw. These sensors are made of microelectromechanical systems (MEMS) gyroscopes, accelerometers, and magnetometers. Usually, they are used in aircraft flight instruments due to their high precision. In the present article, these sensors were used to test safety nets, protecting people or objects falling from heights. The measurement was made for two heights: 6 m and 3.5 m. During the research, a type of mannequin that is a representative model of the human body for the largest segment of the adult population was used. The measurement was carried out using two independent measurement systems. One recorded the accelerations at the chest of the tested object, while the sensors of the second system were placed at the head, arms, and legs. The compiled measurement results were related to the permissible acceleration values that do not threaten human health and life.

## 1. Introduction

### 1.1. Safety Nets

Working at heights involves a risk of falling-especially in industries such as construction, mining, or energy. Both collective and individual protection measures are used in these areas [1]. Collective Fall Protection systems consist of safety nets and guardrails which are designed to protect workers in high-risk areas without requiring specific user knowledge [2]. Several articles on the mitigation category are dedicated to research on fall protection systems, including application limitations, the current state, and the implementation of fall protection and testing of current technologies. Based on research, Zuluaga et al. measured the effectiveness of fall protection systems for bridgework using wearable technology and utility analysis and suggested compatible supplementary devices [3]. In [4], fall protection systems are utilized in the construction of high buildings. This study considered technical aspects regarding the effective installation of fall protection systems. Researchers are increasingly interested in examining aspects of real-time sensing and monitoring technology to address the FFH risk at construction sites. Even though collective protection does not require specific training, personal protection should be coupled with induction training to boost workers’ alertness about hazardous situations [5]. In [6], the causes of accidents were identified from a scientific, theoretical point of view and detailed as follows: lack of training, quality, management, and identification of risks regarding implementing Personal Protective Equipment (PPE). Researchers in [7] observed that fatal accidents from heights can be prevented. Proper planning and education play vital roles in this regard. In PPE and collective protection measures, the acceleration value occurring in a person’s body (in individual body parts) during a fall plays a key role. In [8], the researchers analyzed the causes of falls and reconstructed the courses of many accidents. The researchers demonstrated that this cannot be clearly described and standardized. In [5], the focus was on assistive technologies for fall prevention. The current research emphasizes using high-tech gadgets, analytical modules, and information systems. Emerging virtual reality concepts have changed the approach from reactive to proactive and enabled real-time monitoring of construction activities to improve alertness to fall hazards.

Safety nets are one of the best methods of collective protection when working at heights. They are often used on construction sites, providing economical and convenient transport protection against people or objects falling from heights. Proper installation of nets makes it possible to refrain from using individual fall protection measures. They can replace heavy scaffolding (work platform netting systems) or balustrades. They take up less space and are less demanding for transport. Additionally, they are equipped with an edge rope with which they are attached to the structure [9].

**Figure 1:**
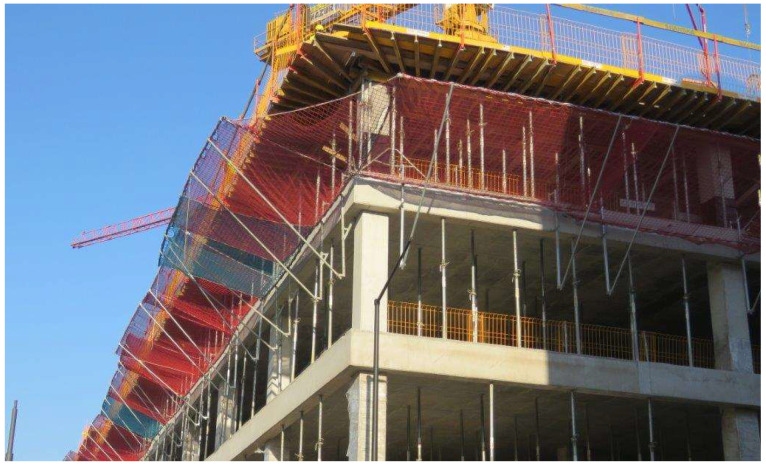
Detailed view of the safety system with safety nets.

**Figure 2:**
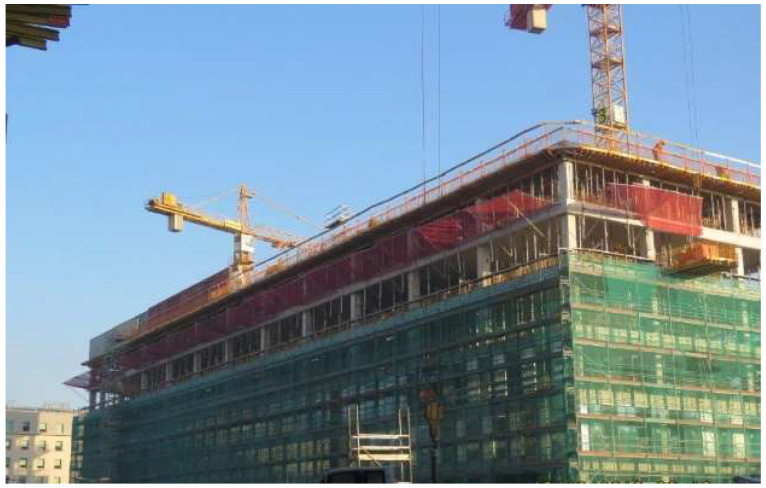
General view of the safety system with safety nets.

Most safety net protection systems are designed and manufactured based on applicable regulations and the EN 1263-1:2015-2 [10] and EN 1263-2:2015-2 [11] standards. The document confirming this compliance is a certificate issued by an independent laboratory. Testing such a net involves performing two drops of a steel ball weighing 100 kg from a height of 7 m. The test is passed if the ball is stopped during the second drop to the same place. In addition, there is a condition that the supporting elements located below the net should be constructed so that a falling person cannot hit them. This condition is challenging to meet because the nets are very flexible. In the event of a fall from a greater height, i.e., 6 m, there is a risk of hitting one’s head against a metal element of the support located near or on the edge of the net. Therefore, tests were carried out from 6 and 3.5 m heights to determine the impact of such a fall arrest. The standard mentioned above allows for protection against falls from a height of 6 m. However, in construction practice, the T system nets are installed in such a way as to protect people working on the floor above. Therefore, the fall height is 3.5 m. This is the standard floor height for office buildings (Figure 1 and Figure 2).

### 1.2. Allowable Accelerations for Humans

Due to the locations of organs with delicate blood supply (brain, eyeballs), the direction of acceleration causes the inflow or outflow of blood, which changes the blood pressure in these organs. In the event of an inflow of blood and an increase in blood pressure, the probability of hemorrhages to the brain, threatening health and life, or to the eyeballs, which may result in partial or permanent impairment of vision, increases. For this reason, the human body tolerates positive overloads much better than negative ones. The highest, yet shortest, g-forces occur during catapulting (even 20–22 g in some Soviet designs, 12–14 g in Western aircraft), competitive aerobatics, and air combat in fighter aircraft (up to 10 g) [12]. High overloads occur during the aircraft’s quick recovery (recovery) from a dive and generally during all air maneuvers while flying at high speeds in an arc. In everyday life, a state of overload can be experienced, for example, in a starting or stopping elevator, in a vehicle that is turning quickly (drifting) or a car overcoming bumps, during solid acceleration and braking in a car, or while traveling by plane—especially when there is turbulence. The greatest overloads occur during road accidents and plane crashes—they usually cause multi-organ injuries. The average person can withstand overloads of up to 5 g. According to [13], persistent human and animal experiments applicable to space flight and crash impact forces are analyzed in this literature survey on human tolerance to rapidly applied accelerations. Human tolerance to sudden acceleration depends on the direction in which the accelerating force is applied to the body, the magnitude of the accelerating force, how long the accelerating force is applied, how rapidly the accelerating force is applied, and how the occupant’s body is supported during acceleration. If a force of 4 to 6 gs is maintained for more than a few seconds, the results could be devastating, such as blackouts to death [14,15].

A typical person can withstand positive/upward loads of up to +5 g, and military aircraft pilots can withstand up to +9 g (temporary). Human resistance to “negative”/“down” overloads is lower, usually −2 g/−3 g [16].

### 1.3. Aim of the Work

It is challenging to take into account the damage to the human body by safety nets. Based on past observations, damage during a fall can be of two types: damage to internal organs and damage to external organs. Damage to internal organs directly threatens health and life, and is most affected by the impact of acceleration. External damage, such as fractures or abrasions to the skin, most often causes only temporary indisposition and is not directly life-threatening. This article focuses on an analysis of the first group of damage, i.e., damage to internal organs. During a fall, individual parts of the human body experience variable accelerations, which are highest at the moment of impact. This article analyzes the accelerations occurring in the human body, i.e., the arms, legs, head and chest, in the event of falls from two heights, 6 m and 3.5 m, onto safety nets. This analysis of permissible overloads and underloads allows us to determine which body parts are most exposed to damage.

## 2. Materials and Methods

### 2.1. Theoretical Basis of the Collision Experiment

The study of the dynamic interaction between a falling person and a trapping net should consider two possibilities: trapping devices with rigidly fixed anchors and nets freely fixed on the frame and trapping devices with pivotally fixed anchors and freely fixed nets. In the first case, brackets are installed so that the bracket angle of deflection remains constant in trapping (brackets’ possible elastic deformations are neglected), and the net is located at some height from the bracket attachment basis. The studies and tests carried out on trapping devices with rigidly fixed brackets and freely hanging or fixed-on-frame nets showed that it is preferable to install brackets for reducing dynamic loads with a maximum possible angle to the horizontal plane with observance of the required condition for ensuring trapping of a falling item [17].

If an item is falling from height H (m) with zero initial vertical velocity, the average body acceleration during braking action, a (m/s^2^), can be evaluated as shown by Equation (1):

(1) $$a=\frac{2gH}{h_{max}}\;$$

where *h_max_* represents the fall dummy’s maximum center of gravity shift and *g* is the gravitational acceleration in m/s^2^ [18]. Based on this, when using trapping devices with rigidly fixed brackets for various falling options, acceleration values may reach about 20 g, which is quite acceptable for falling items which do not cause destruction of the structure and nets of the device, but does not exclude possible injury to person [19].

Four phases can be distinguished in the movement of a falling person:

1. Phase 1—free fall until contact with the net.
2. Phase 2—falling with decreasing acceleration until the gravitational forces and the elastic forces of the mesh are balanced.
3. Phase 3—fall with braking caused by the net force directed upwards (net tension greater than weight).
4. Phase 4—upward movement with decreasing acceleration.

During the first phase, the person moves with gravitational acceleration. Because each part of his body moves identically, the individual parts do not press on each other; the jumper experiences a state of weightlessness. As the elastic force of the net appears, the jumper begins to feel his body weight increase, which becomes normal when the net force (and the resulting acceleration) equals zero. At this point, the jumper’s speed is at the maximum. He then experiences a state of overload. The most significant overload value occurs in the lowest position when the jumper stops moving [20,21].

There is a risk of falling onto the edge of the net. The only effective way to minimize this risk is to increase the surface area of the net (where available), adjust the tension of the rope around the net’s perimeter attached to the anchors, or, as a last resort, adjust the parameters of the net system. By changing the net’s width, the initial angle of the bracket installation can be changed. Such a method of net fastening allows an additional slowdown path of the fall to be obtained.

### 2.2. Tested Object

**Figure 3:**
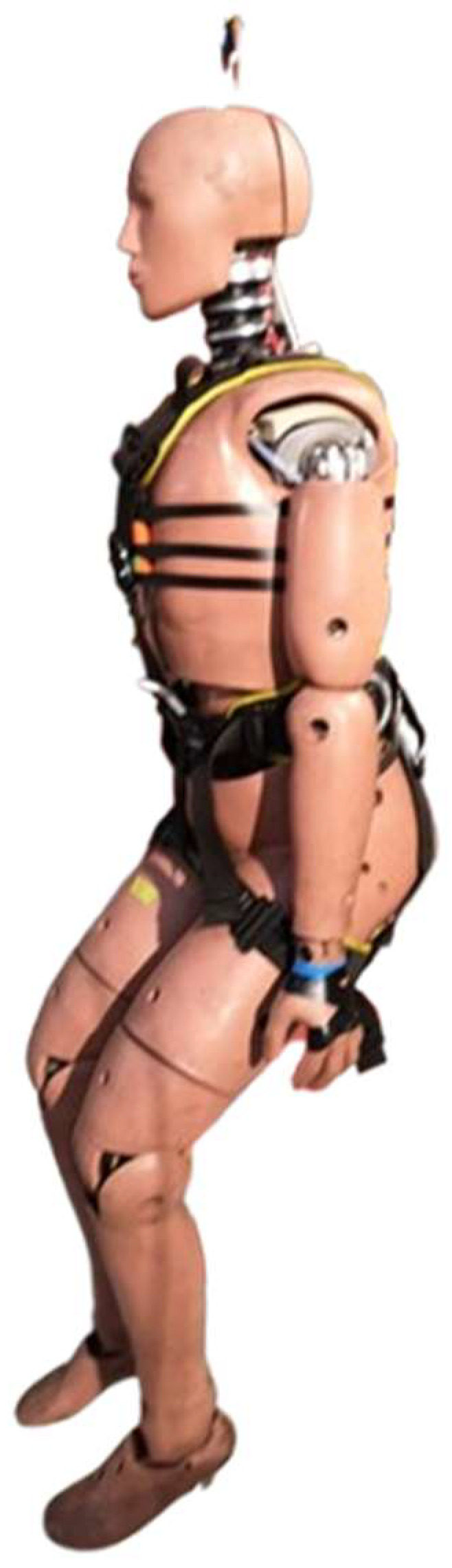
Hybrid III 95th test dummy.

In the research, a Humanetics mannequin Hybrid III 95th Large Male ATD was used (Figure 3), which, based on anthropometric tests, is a representative model of the human body for the largest segment of the adult population [22]. The 95th percentile dummy is used worldwide to evaluate automotive [23] and military safety equipment. The most popular applications are so-called crash tests [24]. The Hybrid III 95 M was initially developed in 1988 by First Technology Safety Systems (then Humanetics) and the Society of Automotive Engineers (SAE) [25]. The Hybrid III 95th Male dummy may be used especially for wheelchair testing, and it is utilized for testing restraint systems in vehicles crash scenarios, but it can be used for other modalities. Its detailed specifications are presented in Table 1 [26].

### 2.3. Equipment and Measurement Method

#### 2.3.1. PRP-W2 Registration System

**Figure 4:**
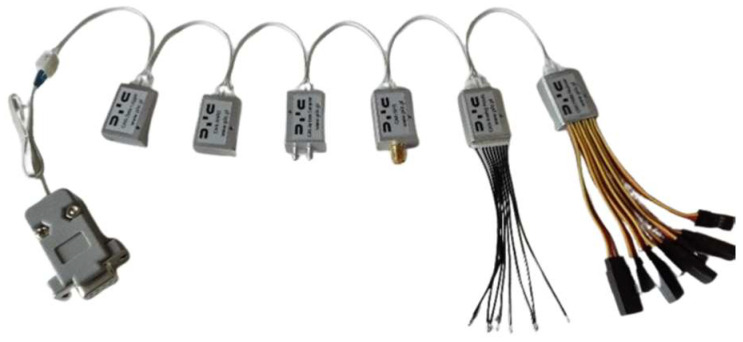
View of a set of modules connected by the CAN bus of the PRP-W2 system. From the left: data recorder, AHRS system, air data computer, GPS module, analogue input module, PWM input module.

The system consists of miniature modules enabling data acquisition from measurements of fundamental physical quantities. Data from individual modules are sent via the CAN bus (Figure 4). Thanks to the modular structure, it is possible to configure the system depending on needs (for data acquisition and control systems). Each module has configurable parameters. This makes connecting them to existing buses (e.g., onboard uncrewed aerial vehicles) possible.

The PRP-W2 system can be expanded to 40 modules connected to the CAN bus; the CAN-Aerospace protocol allows calibrated physical quantities to be registered.

PCDL-01 Data logger

**Figure 5:**
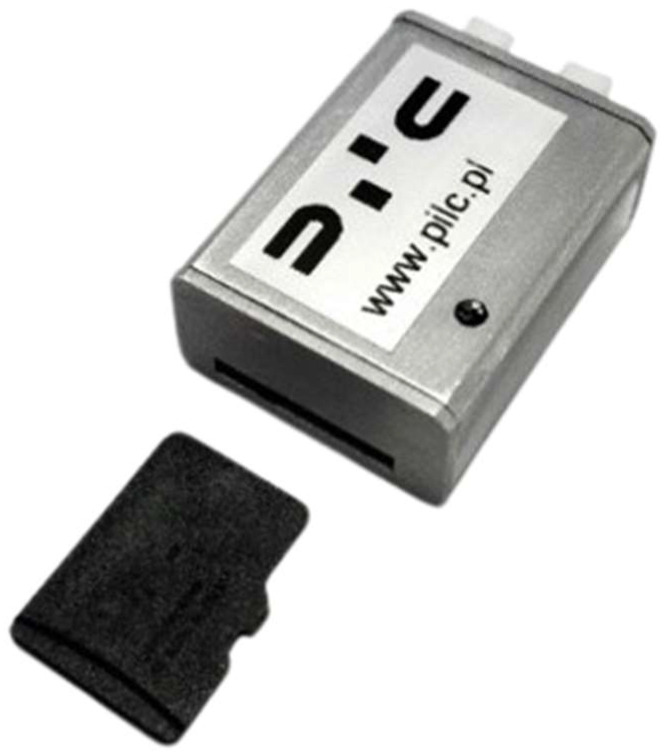
View of the PCDL-01 data recorder.

PCDL-01 (Figure 5) is a miniature data recorder for uncrewed aircraft and other measurement systems. The device is suitable for small- and medium-sized aircraft thanks to its small dimensions, low weight, and low power consumption. The module communicates via the CAN bus using the CAN aerospace protocol. Measurement data are recorded on a microSD card. It is possible to monitor and analyze data via dedicated PC software. The device can be used as a flight test recorder thanks to its durable housing.

PCAH-01 Reference frame

PCAH-01 is a miniature reference system designed mainly for uncrewed aerial vehicles. The device is suitable for small- and medium-sized aircraft thanks to its small dimensions, low weight, and low power consumption. The measurement unit provides information on accelerations, angular velocities, values of the Earth’s magnetic field on all three axes, and spatial orientation angles. Data can be sent at a frequency of up to 1 kHz. The module communicates via the CAN bus using the CAN aerospace protocol.

PCAI-01 Analog input interface

**Figure 6:**
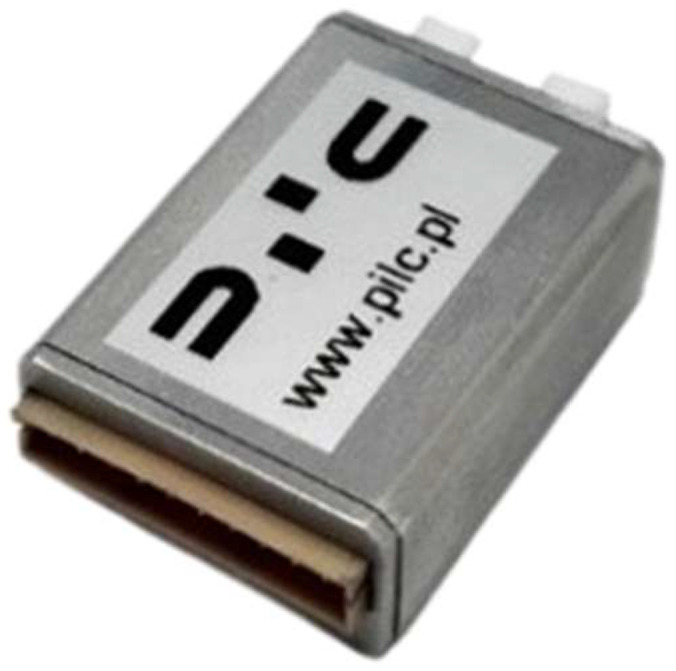
View of the PCAI-01 analogue input module.

PCAI-01 (Figure 6) is an 8-channel miniature interface for measuring voltage signals. The device is suitable for small- and medium-sized aircraft thanks to its small dimensions, low weight, and low power consumption. The module communicates via the CAN bus using the CAN aerospace protocol. Measurement data can be sent at a frequency of up to 1 kHz.

Currently, the system is being expanded to accommodate the telemetric supervision of the experiment.

The AHRS system is a set of transducers containing three-axis accelerometers, three-axis angular velocity transducers, and a three-axis magnetometer. The AHRS system processor determines spatial orientation angles based on recorded output signals from the following transducers:

− PCAI-01 (analogue interface, 2 pcs.).
− Acceleration sensor module (5 pieces were used, located in the arms, legs, and head).

The data acquisition block is the system manufactured by PILC [27]. It is an independent module that measures and records data such as accelerations, angular velocities, spatial orientation angles, barometric height, and measurement time. The data are saved to the SD memory card.

#### 2.3.2. GUARDA Parachutist Flight Recorder

**Figure 7:**
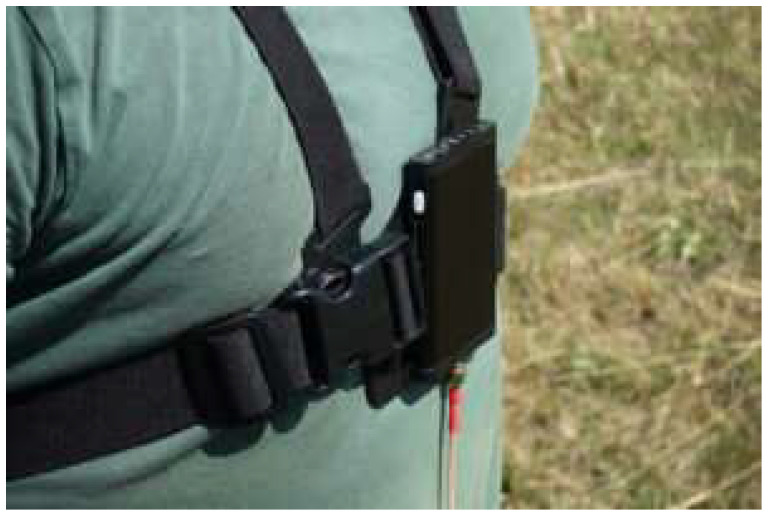
GUARDA recorder placed on the parachute jumper’s chest.

An independent GUARDA recorder was used to redundantly record the drop parameters. This is a unique recorder for testing the flight and drop parameters of parachute jumpers. The system is prototyped by PILC [27]. It was placed on the dummy’s chest, as shown in Figure 7. The GUARDA recorder enables the recording of parameters such as the jumper’s spatial orientation angles during the jump, accelerations acting on him during the canopy’s opening, and touchdown. In [28], it was concluded that fall detection using waist- or head-worn triaxial accelerometers is efficient even with relatively simple threshold-based algorithms. A head-worn accelerometer provides excellent impact detection sensitivity, but confers limitations concerning usability and acceptance. Thus, a triaxial waist-worn accelerometer using an algorithm that recognizes the fall, impact, and posture after the fall might be optimal for fall detection.

List of parameters recorded with the GUARDA recorder, including:

− Barometric altitude;
− Descent speed;
− Geographical position;
− Spatial orientation angles;
− Angular velocities;
− Accelerations acting on the recorder.

Basic recorder parameters:

− Dimensions of 92 × 70 × 12 [mm^3^];
− Weight of 120 g;
− Charging and reading data via USB-C cable.

The metrological parameters of the recorder were equivalent to those of the PRP-W2 system. The data saved in xxx.dat binary files were converted to graphic or text form by the supervisory program described.

The accelerations acting on the limbs and center of gravity of the Hybrid III mannequin were recorded using MEMS accelerometers with voltage output. Voltage measurement and recording on the SD memory card were performed with the PRP-W2 system recorder. The reading and analysis of recorded data was carried out by the program supervising the PRP-W2 system.

### 2.4. Installed Sensors on the Dummy

**Figure 8:**
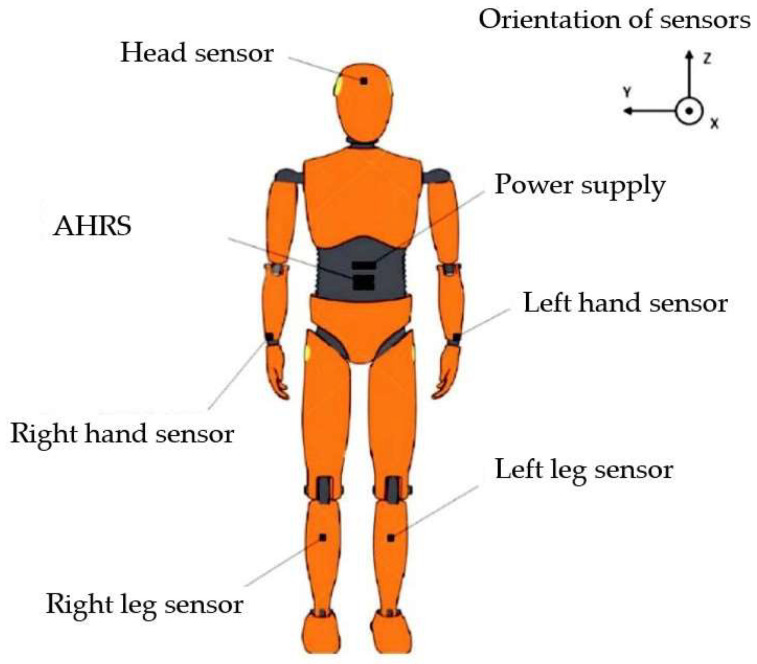
Arrangement of the elements of the PRP-W2 measurement system.

The elements of the PRP-W2 system were mounted on the arms and legs and inside the abdomen and head of the dummy. The elements of the PRP-W2 system were installed before the tests began. The system was mounted so that the accelerometer’s *z* axis coincided with the spine’s line and its Y axis with the line of the right arm extended to the side (Figure 8).

**Figure 9:**
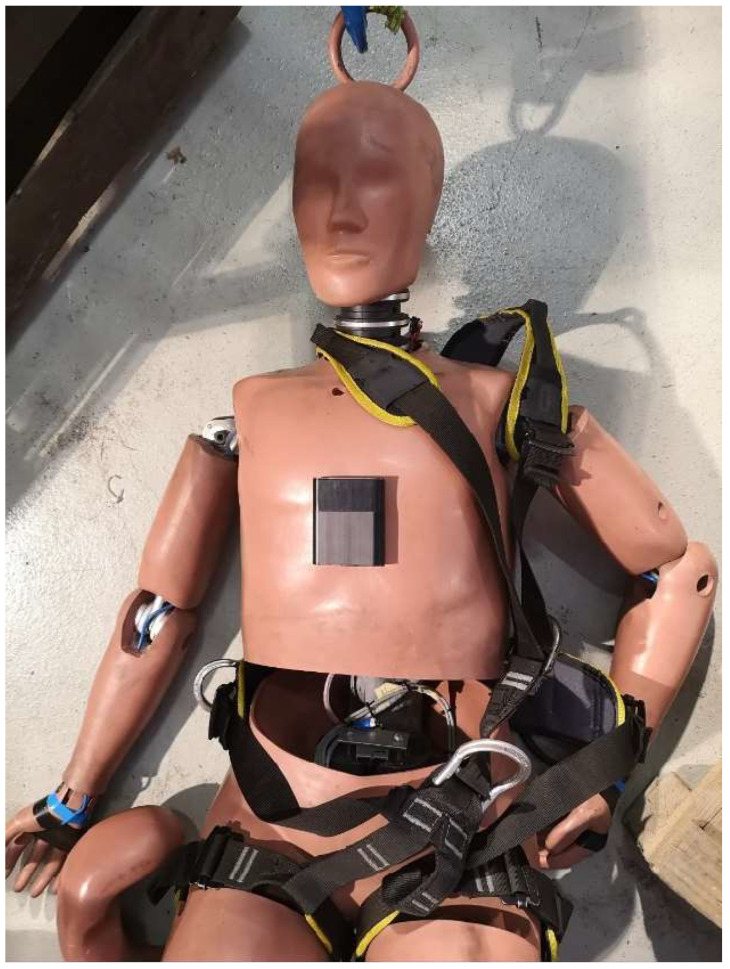
Installation of the GUARDA recorder on the chest.

The GUARDA system is a one-piece module mounted in the center of the chest (Figure 9) when putting the harness on the mannequin before the drop.

The PRP-W2 system is set to record instantaneous accelerations (10 ms sampling is programmed).

## 3. Description of the Research Conducted

**Figure 10:**
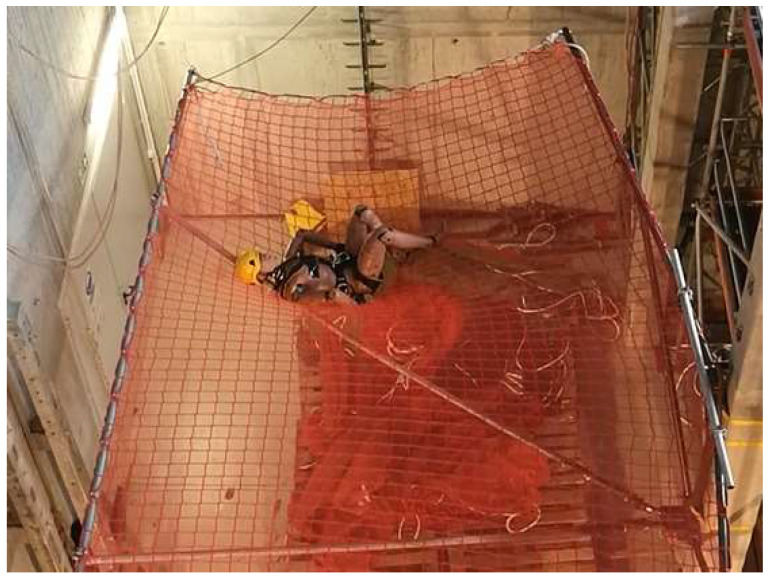
Measurement conditions.

The safety nets used herein were made of high-strength polypropylene with meshes of 10 cm × 10 cm and 4–5 mm thick ropes. During the measurement, an attempt was made to reflect the most likely case of a fall, i.e., when the dummy was in a standing position facing the net. The first drop was made from a height of 6 m (between the lowest point of dummy and the net), and the second was from a height of 3.5 m (between the lowest point of dummy and the net). The net was at a height of 4.3 m above the ground (Figure 10).

The graphs in Section 4 present the instantaneous values of linear accelerations affecting individual human elements.

## 4. Results

The selected sampling rate of the data acquisition system of input signals was limited to a value of 10 ms per channel. This was dictated by the fact that the impact of faster changes on soft tissues (internal organs) does not affect their displacement, so overloads even of large values and durations of about 1 ms do not pose a threat to the human body.

Accelerations were described for all analyzed body parts along the three directions of the rectangular Cartesian system (xyz). The exact directions are shown in Figure 8. On the graphs, it is indicated as *a_i_* [*g*], where the *i* is described by the proper axis (i = *x*, *y*, *z*).

Dump no. 1 (registration using the PRP-W2 system); H = 6.0 m

The mannequin fell onto its back. It fell onto the net, bounced off it and flew slightly to the side, falling outside the net and onto the concrete. The analysis below interprets only the accelerations when the dummy fell onto the net.

**Figure 11:**
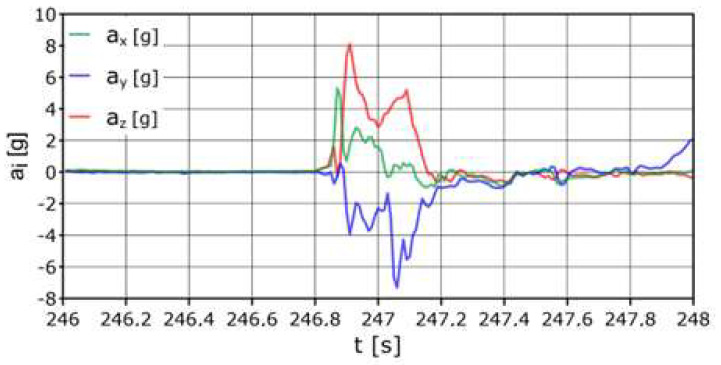
Acceleration variation distribution for the center of gravity.

According to Figure 11, when hitting the net, the maximum acceleration was a_z_ = +8.1 g, and the total acceleration a_z_ was >+5 g in a time not longer than 0.5 s. The longitudinal acceleration had a maximum value a_x_ = +6.7 g (one sample), higher than +2 g in a time not longer than 0.2 s. The recorded maximum lateral acceleration a_y_ = −8.4 g. It was <−2 g for a total of about 0.3 s.

**Figure 12:**
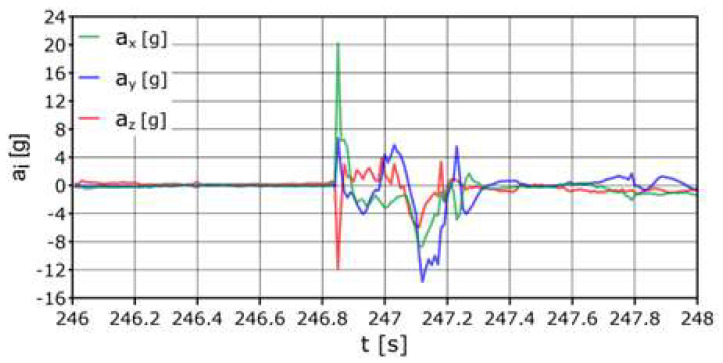
Acceleration variation distribution for the head sensor.

The head was subjected to a maximum acceleration of a_x_~26.5 g in less than 0.02 s (forward movement) (Figure 12). In this direction, the acceleration was a_x_ ≥ 8 g in a time (in total) that was not longer than 0.04 s. There should be no damage to the cervical spine at this stage. However, the negative a_x_ acceleration acted on the head for longer, reaching up to −8.5 g (one sample) negative acceleration. When hitting the net, the following accelerations occurred on the head: a_y_ = +9 g (movement to the right), a_y_ =−4.2 g (movement to the left), a_y_ = +6 g (movement to the right), a_y_ =−14.3 g (movement left), a_y_ = +6.85 (move to the right), and a_y_ =−4 g (move to the left). The described overloads occurred consecutively for a total time of approximately 0.5 s.

The maximum acceleration recorded by the sensor located in the right leg was a_z_ = +12.1 g (movement upwards—towards the body), a_y_ = −19.4 g (and two samples had data showing −13.3 g and −14 g (left movement)), a_x_ = +9.4 g (and +8.4 g (forward movement)). Accelerations on the axles exceeding the range {+3 g:−2 g} occurred within approximately 0.3 s. The sensor in the left leg recorded accelerations >+4 g on the *z* axis in a total time of approximately 0.1 s (the maximum was +13.2 g). This indicates an acceleration in upward movement (towards the body). The acceleration in the lateral axis in the extreme sample was a_y_ =−19.1 g (single sample), which meant the leg moved to the left (a sample with the value a_y_ =−11.9 g was also recorded). Values <−5 g on the *y* axis occurred in a shorter time than 0.1 s. The acceleration on the *x* axis cannot be adequately interpreted—the recorded data indicate a damaged measurement channel. The measured accelerations, recorded by the sensor placed in the left hand, initially reached a_y_ = +26.7 g, a_x_ = +19.2 g, a_z_ = −23.1 g, a_y_ = −16.7 g (accelerations acting successively in the following directions: right, forward, down, and left in a total time not exceeding 0.1 s), and then the accelerations reached values of up to a_y_ = +5 g, a_z_ = +25.9 g, a_y_ = −10, 4 g. The mannequin fell so that the body pressed its right hand against the net. Therefore, no significant hand movement was recorded, as in the case of the left hand. The maximum recorded acceleration of a_x_ = −26.2 g, a_y_ = +15.8 g, a_z_ = +16.3 g. Accelerations on the axes exceeding the range {−2 g:+3 g} occurred at approximately 0.5 s.

Dump no. 2 (registration using the PRP-W2 system); H = 3.5 m

The mannequin fell onto its back. It fell onto the net, and a metal part of the frame held the net directly under the net. After the fall, the dummy did not move noticeably from the point of impact.

**Figure 13:**
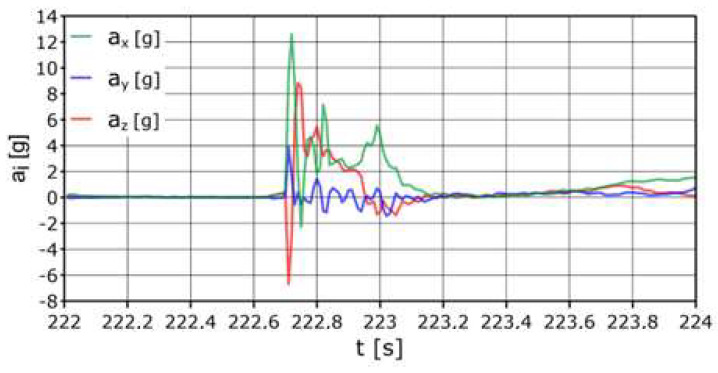
Acceleration variation distribution for the center of gravity.

The AHRS reference system was placed as shown in Figure 8; it was assumed to be the center of gravity of the dummy. The first values recorded directly during the impact were: average acceleration a_z_ = −13.1 g, acceleration longitudinal a_x_ = +16 g, acceleration side a_y_ = +6.8 g. Accelerations on the axes exceeding the range {−2 g:+3 g} occurred within a total duration of approximately 0.3 s (Figure 13).

**Figure 14:**
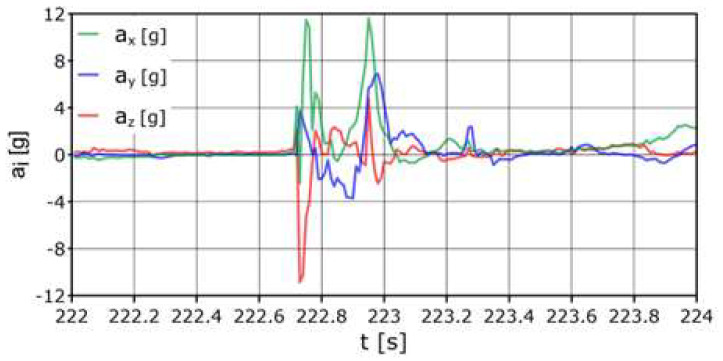
Acceleration variation distribution for the head sensor.

The sensor placed inside the head (Figure 14) recorded accelerations at the limit values of a_x_ = +11.8 g, a_z_ = −15.3 g (movement forward and down (towards the body)). The following sample with a high acceleration value occurred after approximately 0.2 s and amounted to a_x_ = +12.7 g (forward movement). High acceleration values that occurred in a time longer than single milliseconds may (but do not have to) indicate a risk of damage to the cervical spine (compared to dump no. 1, where the acceleration on the *x* axis was twice as high, but the duration was ten times shorter).

The maximum acceleration values recorded by the sensor located in the right leg were a_x_ = +9.9 g (forward movement), a_y_ = −5.2 g, and a_z_ = +11.5 g (acceleration in the “up” direction—towards the body). For the left leg, the values recorded on the *x* axis will not be included due to the damage. The limiting accelerations recorded on the *y* axis were a_y_ = +3 g (movement to the right), a_y_ = −3.4 g (movement to the left), and on the *z* axis a_z_ = +7.3 g (movement up—towards the body). The sensor mounted in the left hand registered accelerations with values of a_x_ = −11.6, a_x_ = +0.6 g, and then a_x_ = +12.9 g (this means that the acceleration acted twice in the “forward” direction, and a slight acceleration value between them means “braking” the hand through the net). On the *y* axis, a maximum acceleration of a_y_ = −12.2 g (movement to the left) was recorded, followed immediately by a_y_ = +19.9 g (movement to the right). On the *z* axis, the acceleration was a_z_ = −24.9 g (downward movement). The maximum accelerations recorded on the right hand were a_x_ = −21.8 g (backward movement), a_y_ = −20.1 g (leftward movement), and a_z_ = −25.9 g (downward movement). After these samples, the acceleration values increased rapidly, which means that the hand moved in the opposite direction.

**Figure 15:**
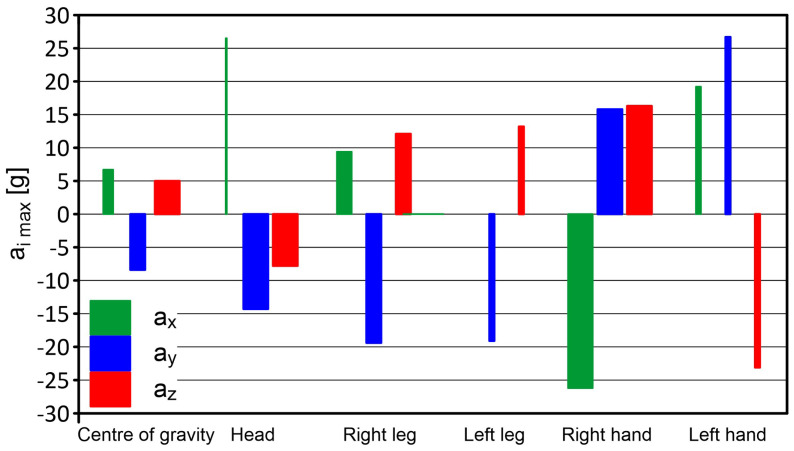
Results of the PRP-W2 system tests—dump no. 1; H = 6.0 m.

**Figure 16:**
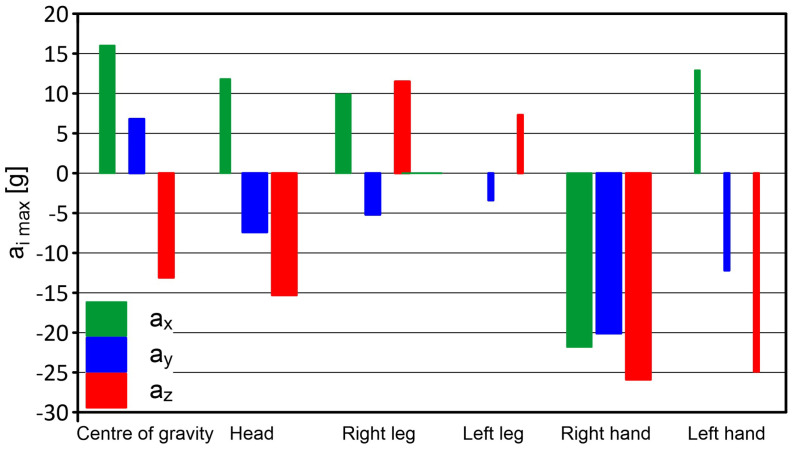
Results of the PRP-W2 system tests—dump no. 2; H = 3.5 m.

The results of the PRP-W2 system tests for two dumps are presented in Table 2 and in Figure 15 and Figure 16. The widths of the bars were determined by the duration of acceleration.

**Figure 17:**
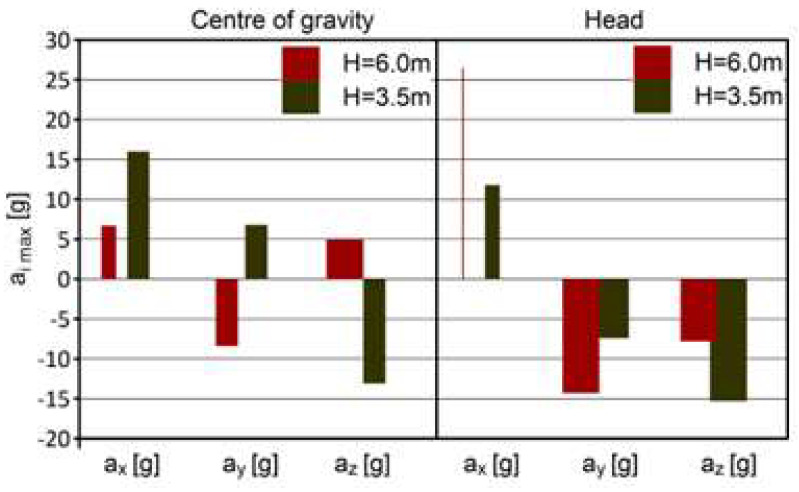
Results of the PRP-W2 system tests—comparison for center of gravity and head.

A comparative analysis was performed for the most exposed organs (center of gravity, head) depending on the fall heights. The results of this analysis are presented in Figure 17.

Registration with the second independent registration system—GUARDA

**Figure 18:**
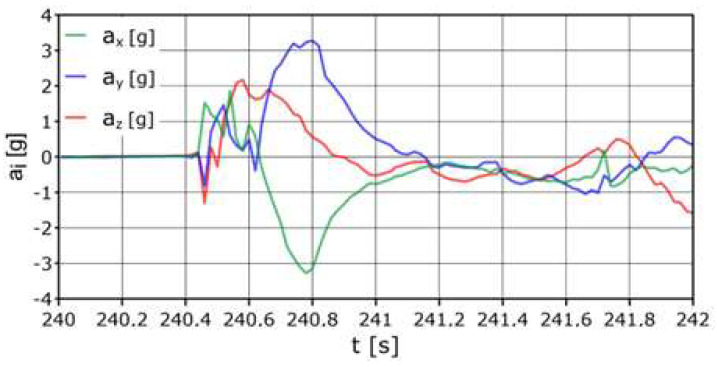
Acceleration variation distribution for the GUARDA system—dump no. 1; H = 6.0 m.

The GUARDA recorder is unique for testing parachute jumpers’ flight and drop parameters. It was placed on the dummy’s chest, as shown in Figure 9. The acceleration variation distribution for the GUARDA system during dump no. 1 is shown in Figure 18.

The maximum values of the recorded accelerations immediately after falling onto the net occurred in 240.779 [s] of the measurement (Figure 18):

1. Normal acceleration a_z_ = 0.551 g;
2. Longitudinal acceleration a_y_ = −3.150 g;
3. Lateral acceleration a_x_ = 3.279 g.

**Figure 19:**
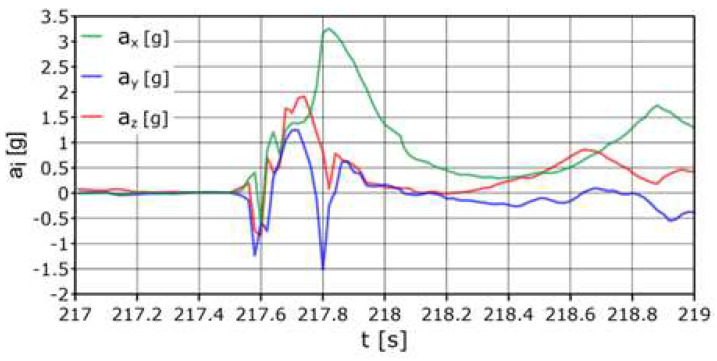
Acceleration variation distribution for the GUARDA system—dump no. 2; H = 3.5 m.

Acceleration variation distribution for the GUARDA system during dump no. 2 is shown in Figure 19.

The maximum values of the recorded accelerations immediately after the fall occurred in 217.811 [s] of the measurement (Figure 19):

1. Normal acceleration *a_z_* = 0.051 g;
2. Longitudinal acceleration *a_y_* = 3.256 g;
3. Lateral acceleration *a_x_* = −0.201 g.

After recording the instantaneous accelerations with the GUARDA recorder, the total accelerations acting on the dummy’s body *a_tot_* were calculated according to Equation (2).

(2) $$a_{tot}=\sqrt{a_{x}^{2}+a_{y}^{2}+a_{z}^{2}}$$

**Figure 20:**
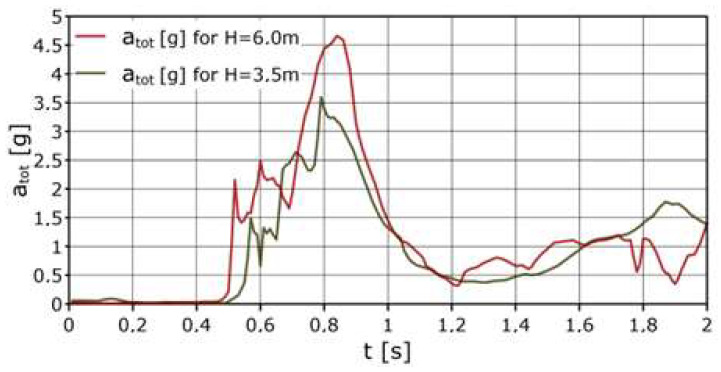
Modulus of acceleration variation a_tot_ distribution for the GUARDA system.

The total accelerations are shown in Figure 20 as the root of the sum of the squares of the accelerations of the *a_x_*, *a_y_*, and *a_z_* components. These are the values occurring during the drops of dummy onto the safety net.

## 5. Discussion

Based on the research carried out, it can be concluded that:

1. Based on the results obtained, it can be concluded that the impact of the fall height impacts both the type of acceleration occurring in the body (overload/underload) and the resulting values.
2. In most of the analyzed cases, for both fall heights, the obtained results are a combination of overloads and underloads along individual axes. The exception is the right hand during the second fall, for which only underload was recorded.
3. The impact of the fall height is most significant for the accelerations occurring in the body for internal organs—the head and the center of gravity. These values increase as the fall height increases. For free body parts (i.e., arms and legs), changing the fall height does not cause a noticeable change; the results are very similar.
4. The change in orientation of the human being is reflected in the accelerations that occur. The highest values appear in the body’s most free parts, i.e., the hands.
5. It is impossible to simulate all possible fall trajectories—in this case, the dummy fell once onto its side and another time onto its back. Depending on the manner of fall, different acceleration values appear in different parts of the body.
6. It is not possible to say unambiguously in which parts of the human body the highest acceleration values (around 20 g) appear. The highest accelerations appeared for the hand-mounted sensors for both of the fall cases presented here. This may be because these body parts were free during the fall.
7. High acceleration values (around 20 g) in the event of a fall also appeared for the head-mounted sensor. However, in contrast to the values appearing in the hands, these were instantaneous overloads, no longer than 2 ms.
8. Simulation tests using the Hybrid 3 dummy do not fully reflect the real values. The mannequin’s mass of approximately 100 kg represents the typical mass of a worker. The primary difference between a dummy and a human body is its greater compliance and flexibility, translating into damping, especially of momentary accelerations (the mannequin is stiff—its “bone” structure is made of metal). It should be assumed that for the actual body of an employee, the values of instantaneous accelerations would be probably much smaller.

## 6. Conclusions

The solution proposed in the article concerns belaying a fall from a height. This solution increases safety and reduces the risk of damage to internal organs, but does not eliminate it. It is an easy-to-fix solution (the frame is just two tubes and a net) with an uncomplicated design. One of the most significant difficulties, for economic and spatial reasons, is to correctly determine the net’s span and minimize the height of a possible fall. Too large a span generates unnecessary costs; too small a span poses a risk of falling out of the net or hitting the frame. The place where the body’s center of gravity falls also plays a key role. Therefore, tube protectors that allow the dummy to move fully onto the grid (assuming that its center of gravity lands on the grid) should be considered in further studies. The streamlined shape of the frame used should also be taken into account. Another risk of the proposed solution is the impact of body parts on the frame. The most sensitive part of the human body, due to its delicate tissues, is the head, which should be further protected. The study also showed that the momentary accelerations acting on the mannequin’s limbs should not endanger the life of a falling worker. The most vulnerable parts are the most accessible parts of the body, i.e., the hands. A limb fracture due to crushing resulting from a change in the spatial orientation of the falling body is very likely. The total acceleration acting on the torso, not exceeding 5 g in 0.5 s, should also not risk damaging the internal organs of a person falling on the grid. Momentary accelerations over 12 g also occurred in this study, but the duration of such overload in the 1–2 ms range is not life-threatening.

## Figures and Tables

**Table 1:** Hybrid III 95th male dummy—detailed specifications.

Overall Dimensions	782.1 × 475.0 × 1389.4 mm^3^
Total weight	101.2 kg
Footprint/work area	782.1 × 475.0 mm^2^
Seated height	919.5 mm

**Table 2:** Results of the PRP-W2 system tests.

Part of Body	Centre of Gravity			Head			Right Leg			Left Leg			Right Hand			Left Hand		
Maximum Acceleration	a_x_ [g]	a_y_ [g]	a_z_ [g]	a_x_ [g]	a_y_ [g]	a_z_ [g]	a_x_ [g]	a_y_ [g]	a_z_ [g]	a_x_ [g]	a_y_ [g]	a_z_ [g]	a_x_ [g]	a_y_ [g]	a_z_ [g]	a_x_ [g]	a_y_ [g]	a_z_ [g]
Duration of Acceleration	t_max_ [s]	t_max_ [s]	t_max_ [s]	t_max_ [s]	t_max_ [s]	t_max_ [s]	t_max_ [s]	t_max_ [s]	t_max_ [s]	t_max_ [s]	t_max_ [s]	t_max_ [s]	t_max_ [s]	t_max_ [s]	t_max_ [s]	t_max_ [s]	t_max_ [s]	t_max_ [s]
Dump no. 1 H = 6.0 m	+6.7	−8.4	+5	+26.5	−14.3	−7.8	+9.4	−19.4	+12.1	-	−19.1	+13.2	−26.2	+15.8	+16.3	+19.2	+26.7	−23.1
	0.2	0.3	0.5	0.02	0.5	0.5	0.3	0.3	0.3	-	0.1	0.1	0.5	0.5	0.5	0.1	0.1	0.1
Dump no. 2 H = 3.5 m	+16	+6.8	−13.1	+11.8	−7.4	−15.3	+9.9	−5.2	+11.5	-	−3.4	+7.3	−21.8	−20.1	−25.9	+12.9	−12.2	−24.9
	0.3	0.3	0.3	0.2	0.5	0.5	0.3	0.3	0.3	-	0.1	0.1	0.5	0.5	0.5	0.1	0,1	0.1

## Data Availability

The original contributions presented in this study are included in the article/Supplementary Materials.

## Supplementary Materials

The following supporting information can be downloaded at: https://www.mdpi.com/article/10.3390/s24237431/s1.
