# Supplementary material for: Use of Attitude and Heading Reference System (AHRS) to Analyze the Impact of Safety Nets on the Accelerations Occurring in the Human Body During a Collision
Source: Sensors (Basel). 2024 Nov 21;24(23):7431. doi: 10.3390/s24237431 (PMC11644487; doi:10.3390/s24237431)
Supplement: Supplementary file 1 [file sensors-24-07431-s001.zip › sensors-3248622-supplementary.pdf]

sensors

MDPI

Supplementary Material

Use of Attitude and Heading Reference System (AHRS) to Analyze the Impact of Safety Nets on the Accelerations Occurring in the Human Body During a Collision

Mariusz Gołkowski [1], Jerzy Kwaśniewski [2], Maciej Roskosz [2], Paweł Mazurek [2,*], Szymon Molski [2] and Józef Grzybowski [3]

1 CBR Rock Master, 30-079 Krakow, Poland; mariusz.golkowski@rockmaster.eu
2 Department of Machinery Engineering and Transport, Faculty of Mechanical Engineering and Robotics, AGH University of Krakow, 30-059 Krakow, Poland; kwasniew@agh.edu.pl (J.K.); mroskosz@agh.edu.pl (M.R.); molski@agh.edu.pl (S.M.)
3 Department of Avionics and Control, Rzeszów University of Technology, Al, Powstańców Warszawy 8, 35–959 Rzeszów, Poland; aviog@prz.edu.pl
* Correspondence: pmazurek@agh.edu.pl

Dump no. 1 (registration using the PRP-W2 system)

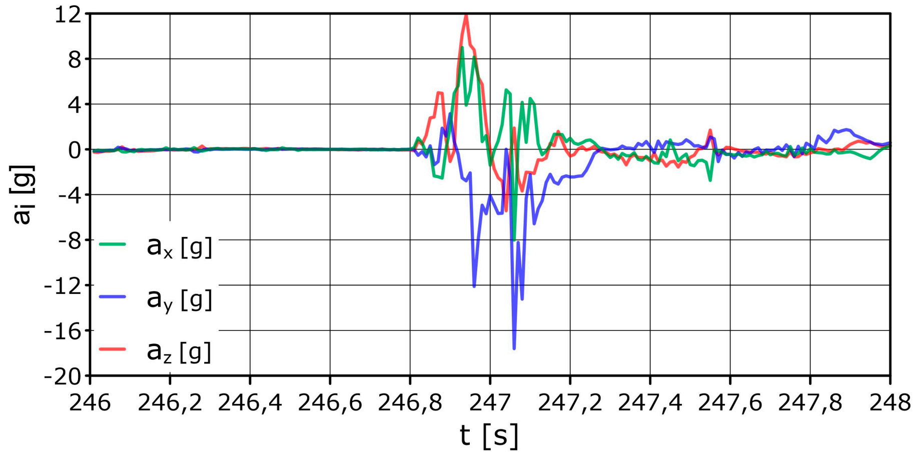

Figure S1. Acceleration variation distribution for the right leg sensor.

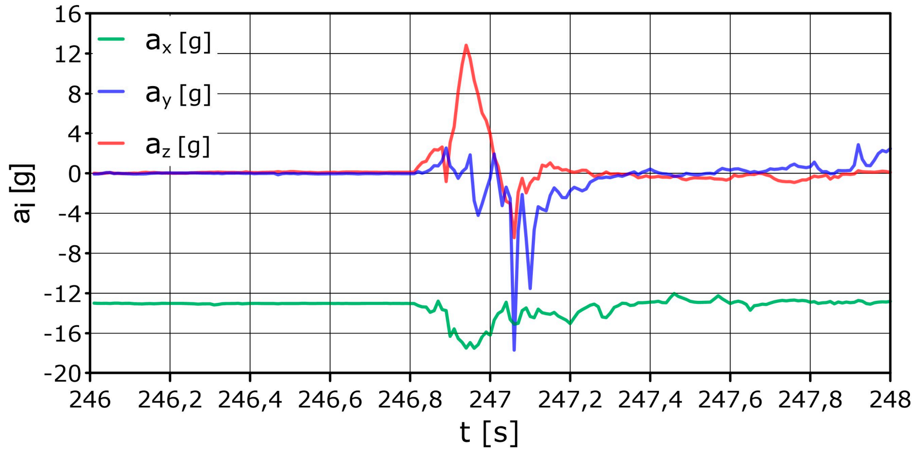

Figure S2. Acceleration variation distribution for the left leg sensor.

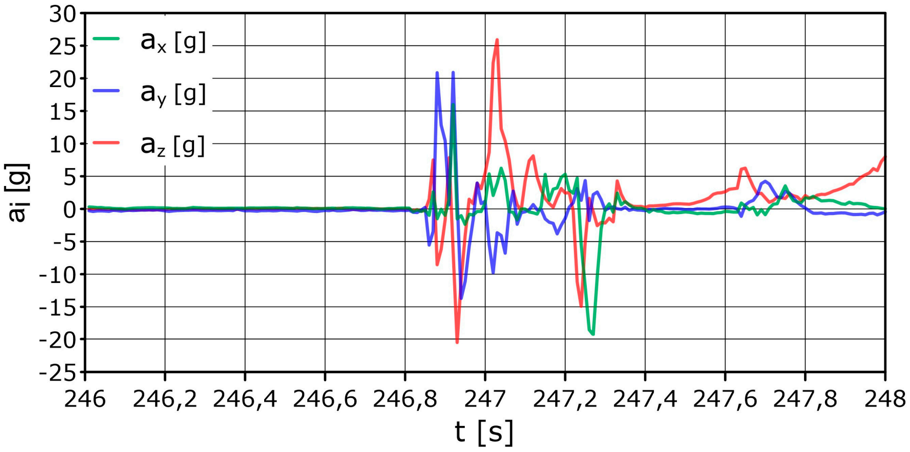

Figure S3. Acceleration variation distribution for the left hand sensor.

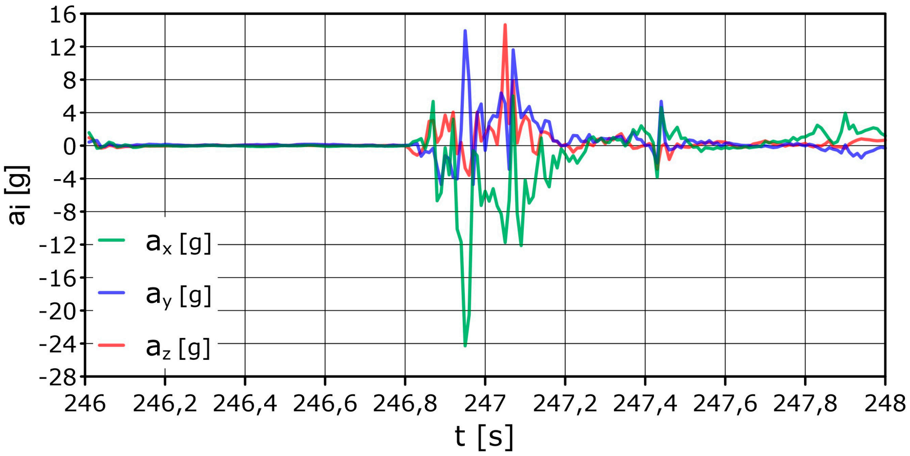

Figure S4. Acceleration variation distribution for the right hand sensor.

Dump no. 2 (registration using the PRP-W2 system)

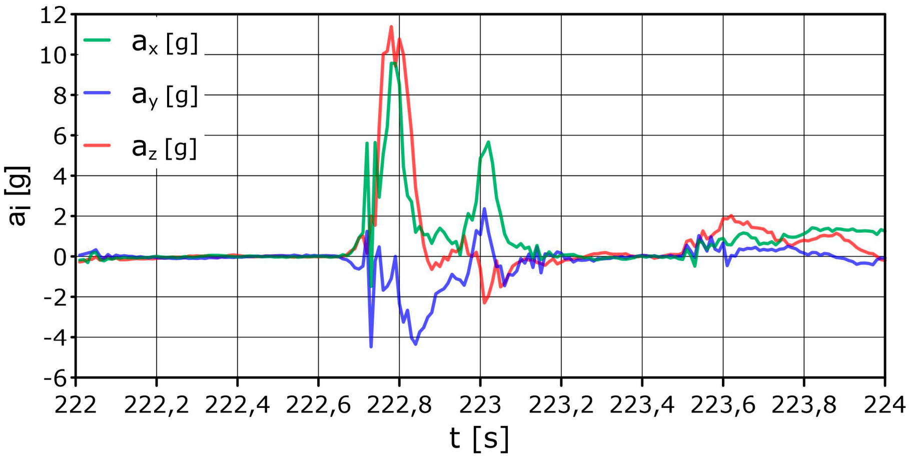

Figure S5. Acceleration variation distribution for the right leg sensor.

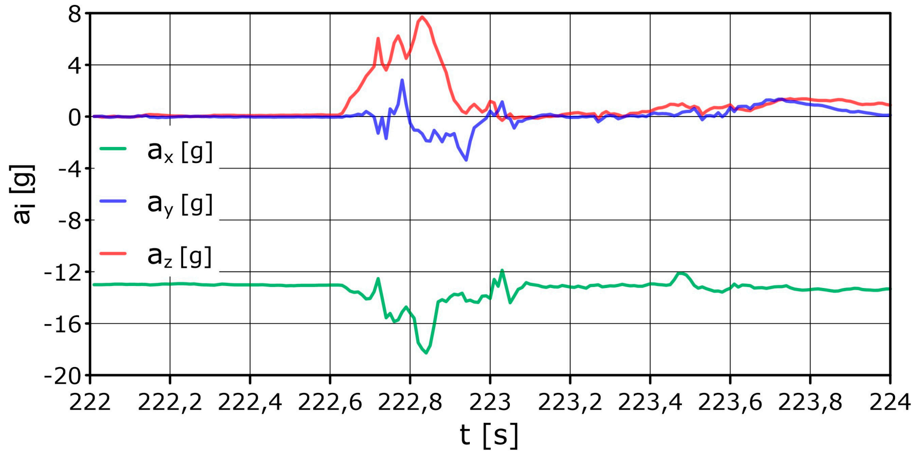

Figure S6. Acceleration variation distribution for the left leg sensor.

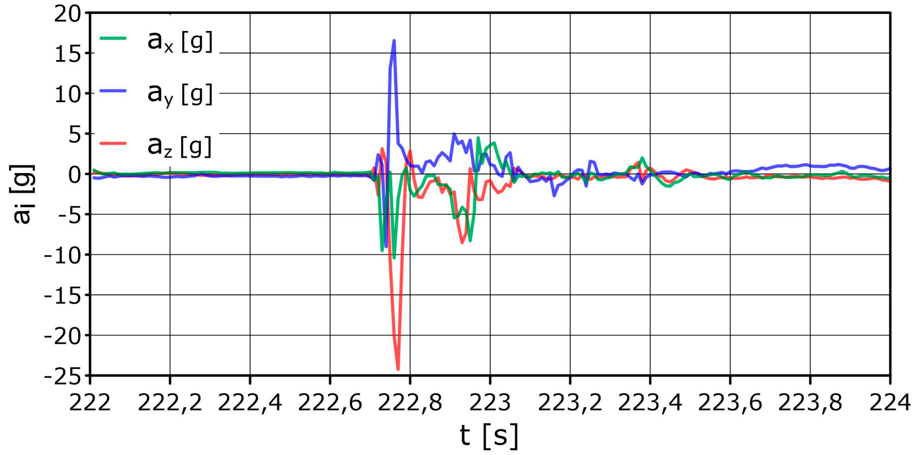

Figure S7. Acceleration variation distribution for the left hand sensor.

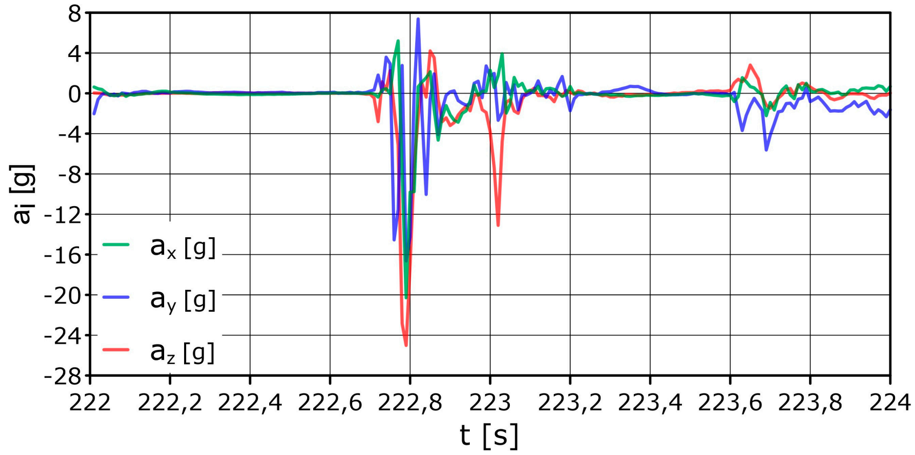

Figure S8. Acceleration variation distribution for the right hand sensor.
